# Novel drug delivery systems targeting oxidative stress in chronic obstructive pulmonary disease: a review

**DOI:** 10.1186/s12951-020-00703-5

**Published:** 2020-10-19

**Authors:** You Xu, Hongmei Liu, Lei Song

**Affiliations:** 1 Department of Respiratory Medicine, Key Laboratory of Organ Regeneration & Transplantation of the Ministry of Education, The First Hospital of Jilin University, Changchun, 130061 People’s Republic of China; 2 Department of Pharmacy, Faculty of Health & Medical Sciences, University of Copenhagen, 2100 Copenhagen, Denmark

**Keywords:** Oxidative stress, Chronic obstructive pulmonary disease, Drug delivery, Nanoparticles, Microparticles, Nanocomposite microparticles

## Abstract

Oxidative stress is significantly involved in the pathogenesis and progression of chronic obstructive pulmonary disease (COPD). Combining antioxidant drugs or nutrients results in a noteworthy therapeutic value in animal models of COPD. However, the benefits have not been reproduced in clinical applications, this may be attributed to the limited absorption, concentration, and half-life of exogenous antioxidants. Therefore, novel drug delivery systems to combat oxidative stress in COPD are needed. This review presents a brief insight into the current knowledge on the role of oxidative stress and highlights the recent trends in novel drug delivery carriers that could aid in combating oxidative stress in COPD. The introduction of nanotechnology has enabled researchers to overcome several problems and improve the pharmacokinetics and bioavailability of drugs. Large porous microparticles, and porous nanoparticle-encapsulated microparticles are the most promising carriers for achieving effective pulmonary deposition of inhaled medication and obtaining controlled drug release. However, translating drug delivery systems for administration in pulmonary clinical settings is still in its initial phases.
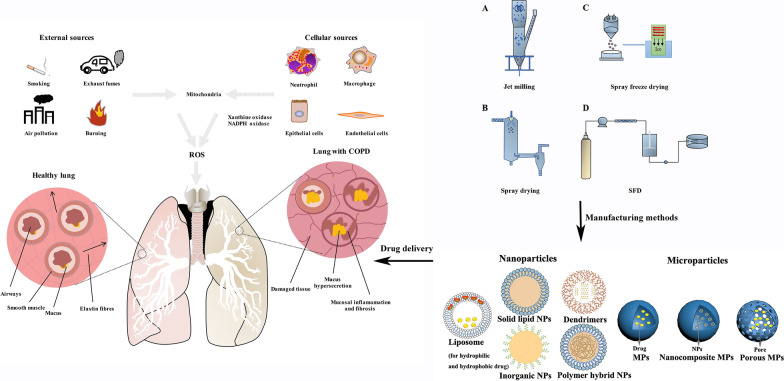

## Introduction

The respiratory tract is constantly exposed to multiple endogenous and exogenous oxidants and develops a series of defence mechanisms to limit the oxidative damage. Oxidative stress, caused by an imbalance between increased oxidative burden and the defective antioxidant system, is involved in cellular and tissue damage related to the pathogenesis and progression of many acute and chronic respiratory diseases including chronic obstructive pulmonary disease (COPD) [1, 2]. COPD is characterised by chronic bronchitis and emphysema, which present with that feature persistent airway inflammation, ultimately leading to a gradual progression of irreversible airway obstruction. It has become a global health problem ranked as the fourth leading cause of death worldwide. There were up to 2.8 million deaths from COPD in 2010 globally and about 175 million patients were suffering from this disease in 2015 [3]. Although therapeutic strategies have advanced to help ease symptoms and prevent complications, there is no cure for COPD. Therefore, new approaches are urgently needed to slow or even stop the progression of this disease and reduce the mortality.

The presence of excessive reactive oxygen species (ROS) in the airway plays a key driving role in the pathogenesis and progression of COPD [4, 5]. Cigarette smoke and biomass fuel-induced oxidative and aldehyde/carbonyl stress are closely associated with the progression and exacerbation of COPD. The oxidant generates airway inflammation that leads to the production of proinflammatory cytokines [6, 7]. It also recruits inflammatory cells such as neutrophils, eosinophils, lymphocytes, and macrophages by potentiating the action of histamine, which injures lung tissues and causes inflammation [8, 9]. Targeting systemic and local oxidative stress using antioxidants/redox modulating agents, or boosting the endogenous levels of antioxidants are strategies for the treatment and management of COPD.

Traditional pharmacotherapy for drug delivery to the lungs can be classified according to the type of therapeutic agents [10, 11]. Unfortunately, COPD cannot be completely cured using pharmacotherapy alone owing to biological barriers, low drug bioavailability and associated safety concerns [12, 13]. Advanced strategies for novel drug delivery systems (DDS) have recently demonstrated promising results as a targeted drug delivery pharmacotherapy. Based on their innate physical properties, novel DDS can improve the pharmacokinetics of the loaded therapeutics and target cells to minimise the adverse effects of drugs [11, 14].

Reviews related to the importance and use of nanoparticles for respiratory diseases are available [15]; However, a comprehensive summary of novel drug delivery systems has not been presented as yet. Domej et al. reviewed the general relevance of free radicals in the development and progression of both COPD and pulmonary emphysema as well as novel perspectives on therapeutic options. The review is very useful when selecting drugs to treat of COPD, but not while selecting delivery systems [16]. Other reviews exploring novel delivery systems related to oxidative stress are not mainly focused on chronic respiratory diseases but not COPD [17]. Owing to the particular pathogenesis and progression of chronic respiratory diseases, the delivery systems for COPD will, by necessity, need to be different from those developed for other lung diseases. A comprehensive review that introduces the role of delivery systems in the treatment of oxidative stress is very important. Therefore, in this review, we discussed the role of oxidative stress and the use of novel delivery systems in the treatment of oxidative and airway inflammation.

### Redox systems in the lung

**Fig. 1:**
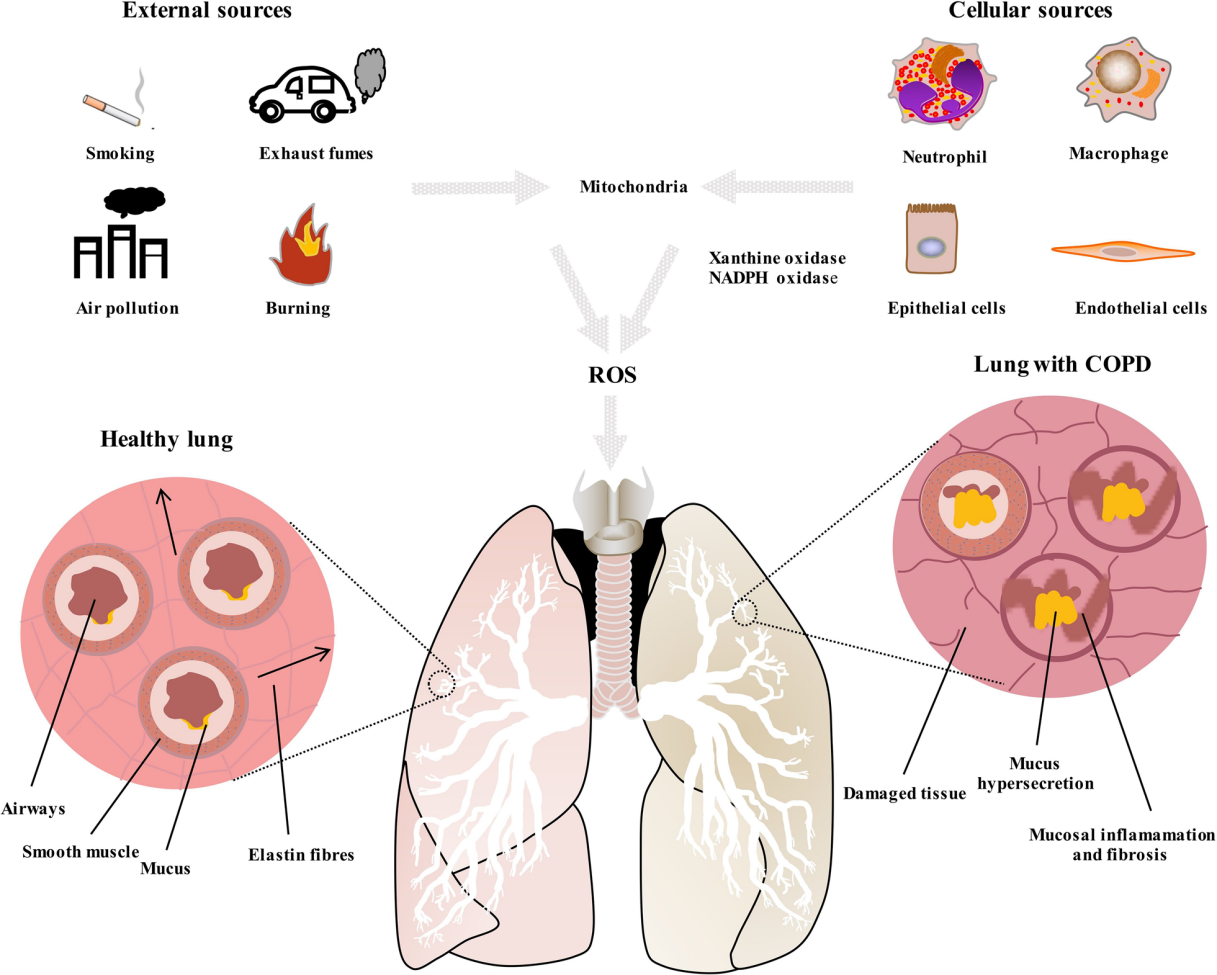
Sources of ROS and differences between a healthy lung and a lung with COPD

Under physiological conditions, ROS and reactive nitrogen species (RNS) can be naturally generated intracellularly by mitochondrial respiration, xanthine/xanthine oxidase, or NADPH oxidase system [18]. Xanthine/xanthine oxidase and the NADPH oxidase system are mainly located in phagocytes and epithelial cells [19]. In response to pathological stimuli, such as viral or bacterial infections, chemical and mechanical factors, phagocytes and endothelial cells in the airways rapidly release large amounts of superoxide anion (O^−^_2_) and hydrogen peroxide (H_2_O_2_), which is referred to as an oxidative burst. The highly specialized function of lungs facilitates oxygen exchange and is, thus, constantly exposed to exogenous ROS, such as cigarette smoke, industrial air pollutants, and airborne pollutants. Tobacco smoke is a mixture of over 4700 chemical compounds including oxidants, which are present at high concentration [20]. Industrial and other airborne pollutants are also sources of oxidants that could be responsible for increased prevalence of COPD in non-smokers [21]. In previous studies, we found that particulate matter (PM) with an aerodynamic diameter ≤ 2.5 μm (PM2.5) significantly induced ROS generation through epigenetic regulation, leading to airway epithelial cell injury and aberrant inflammatory response [22, 23]. The sources of ROS and their influence can be seem in Fig. 1.

ROS modulate the function of all classes of biomolecular, targeting almost all substrates in the cell. Lipids are the most susceptible to oxidation, which results in lipid peroxidation [24]. Lipid peroxidation products such as malondialdehyde, 4-hydroxynonenal and isoprostanes, impair membrane function, inactivate membrane-bound receptors, and increase tissue permeability. ROS can act on proteins to cause side-chain oxidation, backbone fragmentation, unfolding, and misfolding, all resulting in loss of activity [25]. ROS can also damage nucleic acids and cause DNA–protein crosslinking, strand breaking, and alteration in purine and pyridine bass structures, resulting in DNA mutations [26]. The marker of DNA damage is represented by 8-hydroxydeoxyguanosine. These harmful actions lead to different forms of lung injury such as tissue damage, cell activation, and proliferation. Additionally, the damage of the mitochondrial membranes and protein structure can enhance the generation of ROS and lead to DNA impairment and cell death by apoptosis [27].

In healthy lungs, antioxidants, such as catalase (CAT), superoxide dismutase (SOD) and glutathione peroxidase (GPx) provide an endogenous biological defence against cellular or organ injury caused by ROS. These antioxidant genes are predominantly regulated by nuclear factor-erythroid 2-related factor 2 (Nrf2) signalling, which plays a key role in sensing and defying oxidative stress [28]. As a transcription factor, Nrf2 is generally located in the cytoplasm by forming a heterodimer with its repressor Kelch-like ECH-associated protein 1 under physiological condition. In response to oxidative stress, Nrf2 is liberated from the inactive state and then translocated into the nucleus, promoting the expression of antioxidant enzymes by binding to the antioxidant response element [28]. The normal function of Nrf2 signalling is crucial for the maintenance of physiological processes in the lungs. Experiments in Nrf2 knockout mice show enhanced ROS accumulation, inflammatory response, cellular injury in the airway, and increased sensitivity to physical or chemical stimuli [29–32]. Defective Nrf2 signalling was determined in COPD patients and correlated with a decline in lung function and cigarette pack-year [33, 34]. Data from clinical and basic research have identified the protective effects of Nrf2 on COPD. For instance, Nrf2 restored corticosteroid sensitivity of peripheral blood mononuclear cells from COPD patients and, human monocytic U937 cells exposed to cigarette smoke extract [35]. Recent findings on the mechanisms of Nrf2-mediated lung protection in COPD have been reviewed by Barnes [36]. Although it is difficult to develop specific and effective Nrf2 agonist, drugs and strategies targeting Nrf2 signalling are being considered in the treatment of COPD.

### Mechanisms and damaging effects of oxidative stress in COPD

Oxidative stress is now recognised as a major predisposing factor in the pathogenesis of COPD and an excellent target for COPD therapies [37]. Oxidative stress involved in the pathogenesis of COPD is not only the result of the increased burden of oxidants but also owing to a decrease in antioxidative potential [38]. There is clear evidence of increased oxidative burden in patients with COPD. Increased levels of free-radical biomarkers have been detected in the epithelial lining fluid, breath and urine of cigarette smokers and patients with COPD [39–41]. The levels of H_2_O_2_ and arachidonic acid is found to be markedly increased in the exhaled breath condensate of COPD relative to those of the healthy controls [42–44]. Oxidative stress involves several mechanisms that result in the obstructive symptoms observed in COPD (Table 1).

**Table 1:** Main mechanisms of oxidative-burden related COPD

Mechanism	Outcome	Refs.
Imbalance of biological molecules	Reduced antioxidant and antiprotease enzyme activity (SOD, CAT, GPx, α1 antitrypsin); Altered expression of ROS-related enzymes (decreased in CAT, GPx, SOD, and increase in iNOS); Activation of metallo proteinases	[45–49]
Intracellular signaling	Altered expression of ROS related enzymes (decreased in CAT, GPx, SOD, and increase in iNOS)	[50, 51]
Mitochondrial respiration	Altered mitochondrial function	[46, 48, 52, 53]
Upregulation of gene transcription (NF-κB and, AP-1) and increase in cellular cytokine production	Increased gene expression of inflammatory mediators and cytokines (IL-1, TNF-α, IL-8, GM-CSF, iNOS)	[54]
Nuclear histone acetylation/deacetylation balance	Decreased HDAC2 activity and protein expression chronic inflammation (chronic remodelling)	[55, 56]
Remodelling of DNA/chromatin	Decreased histone deacetylase 2	[57]

iNOS, inducible nitric oxide synthase isoenzymes; SLPI, secretory leukocyte protease inhibitor; α1AT, alpha-1-antitrypsin; IL-1, interleukin 1; NF-κB, nuclear factor kappa B; IL-8, interleukin-8; GM-CSF, TNF-α, tumor necrosis factor-α; granulocyte macrophage colony-stimulating factor; iNOS, inducible nitric oxide synthase; HDAC: histone deacetylase

**Fig. 2:**
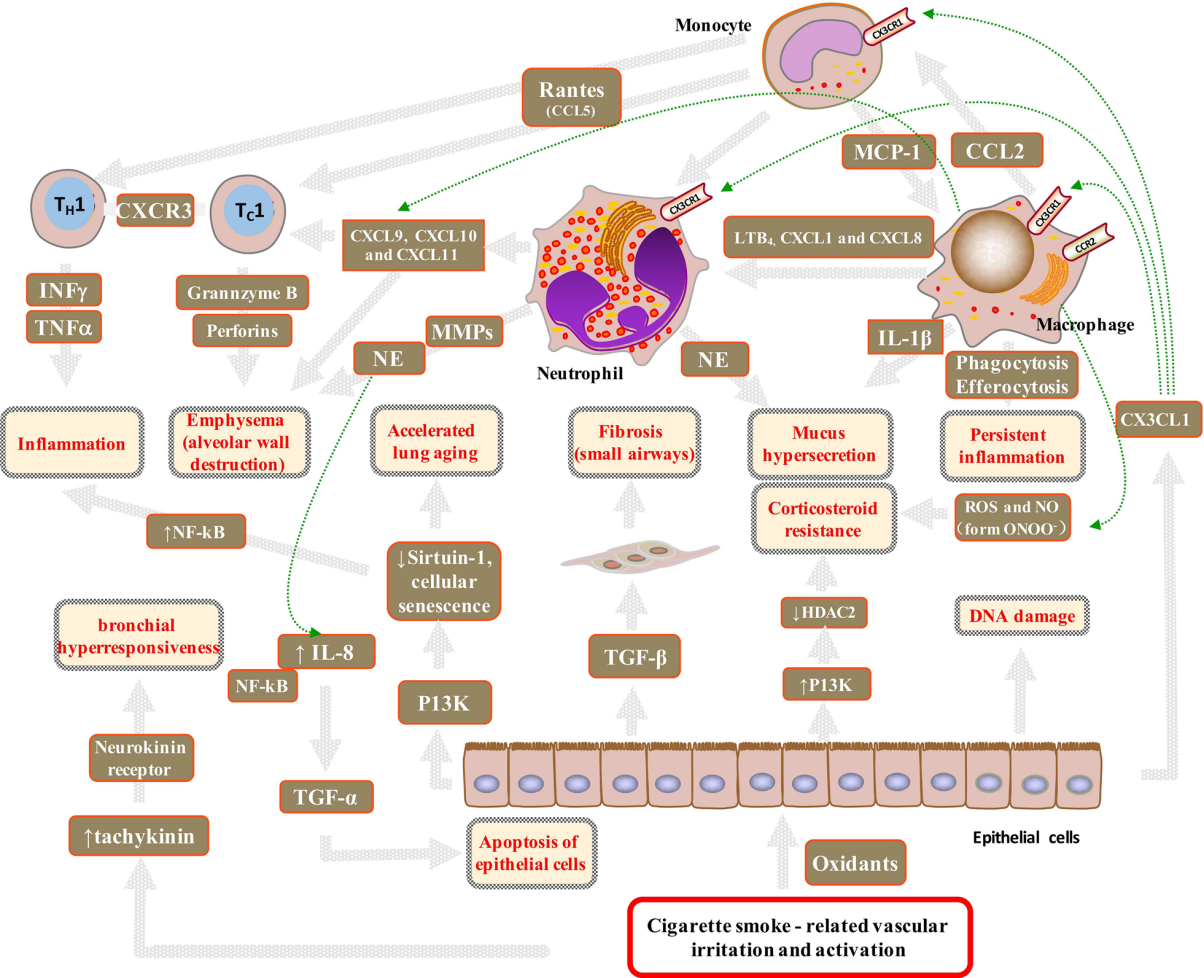
Cigarette smoke-related vascular irritation and activation in patients with COPD. Oxidative irritants including tobacco smoke have a direct effect on the epithelial cells, macrophages, and oedematous basal membranes. The airway smooth muscle markedly increased under excess exposure to ROS. Irritants activate macrophages to release several chemotactic factors including CCL2, which attract inflammatory cells to the lungs and acts on CCR2 to attract monocytes. LTB, CXCL1, and CXCL8 act on CCR2 to attract neutrophils. Neutrophil elastase causes hypersecretion of mucus. CXCL9, CXCL10, and CXCL11 act on CXCR 3, which attracts T_H_1 and T_C_ 1 cells. T_C_1 cells, through the release of perforin and granzyme B, induce apoptosis of type I pneumocytes, thereby contributing to emphysema. IFNγ and TNFα released by T_H_1 stimulate inflammation. Activated macrophages provide oxidative signalling to neutrophils and T_H_1 cells, which cause an imbalance in the protease/antiprotease system and overexpression of MMP-9 and Ne. The increased expression of Ne induces TNF-α by the NF-κB signalling pathway and accelerates apoptosis of epithelial cells. Oxidative stress activates P13K leading to the phosphorylation and inactivation of HDAC2. Macrophages generate ROS and NO that form ONOO-peroxynitrite and might also inhibit the activity of HDAC2. These modifications of HDAC2 result in corticosteroid resistance in COPD patients. Oxidative stress also drives accelerated ageing through the activation of P13K and reduction in sirtuin-1 levels, which leads to cellular senescence and release of inflammatory proteins, further increasing oxidative stress. ROS can induce tachykinin release from the capsaicin-sensitive sensory that acts on the neurokinin receptors in the airways to induce bronchial hyperresponsiveness. P13K, phosphoinositide 3-kinase; IL, interleukin; TGF, transforming growth factor; MMP, matrix metalloproteinase; MUC5AC; Ne, neutrophil elastase; ROS, reactive oxygen species; CCL, CC-chemokine ligand; CCR, CC-chemokine receptor; CXCL, CXC-chemokine ligand; CXCR, CXC-chemokine receptor; T_H_1, T helper1 cells; T_C_1, type 1 cytotoxic; NO, nitric oxide; ONOO^−^, peroxynitrite

The pathogenesis of COPD involves several pathogenetic processes including oxidative stress, inflammation, protease/antiprotease imbalance, apoptosis, and cellular senescence [43, 58] (Fig. 2); however, the relative contribution of each of these pathologies to COPD varies among patients.

The major pathological features of COPD are obstructive bronchiolitis, emphysema, and mucus hypersecretion [59]. Oxidative stress might result the activation of the proinflammatory transcription factor, NF-κB, impaired antiprotease defences, cellular senescence, and corticosteroid resistance. Oxidative stress can impair the function of antiproteases, such as α1-antitrypsin and secretory leukoprotease inhibitor, thereby accelerating the breakdown of elastin in the lung parenchyma. Oxidative stress accelerates lung ageing because of the defective function of endogenous antiageing molecules such as sirtuins, which have a role in genomic stability and can protect against ageing [60]. The reduced activity and expression of sirtuin-1 eventually leads to cellular senescence. Senescent cells also release generate inflammatory proteins including TNF-α, IL-1, IL-6, CXCL8, CCL2, and MMPs [61]. Corticosteroid resistance is caused by an imbalance in the expression of the proinflammatory enzymes in COPD patients. The damage caused by oxidative stress increases proinflammatory enzyme expression in COPD patients via a decrease in HDAC activity [62, 63] and an increase in histone acetyltransferase activity [6]. Therefore, patients with COPD respond poorly to corticosteroid treatment, which fails to suppress inflammation even in high doses of inhaled or oral corticosteroids are used [64]. Additionally, oxidative stress can reduce anti-inflammatory defence, such as CAT activity, which has been demonstrated to be significantly reduced in patients with COPD [48, 49]. The levels of serum nitro-tyrosine [39] and lipid-peroxidation products increase in patients with COPD, resulting in increased systemic inflammation via the production of proinflammatory cytokines, ultimately causing cachexia [65] and thromboembolic events [66].

Oxidative stress also causes small-airway fibrosis. Epithelial cells are activated by cigarette smoking-related vascular irritation and activation and result in the production of inflammatory mediators [67]. Epithelial cells in the small airways express TGF-β, which induces local fibrosis and narrowing of small airways. Oxidative stress causes airflow obstruction that leads to tissue hypoxia and could stimulate an inflammatory response [68]. It can also damage and cause apoptosis of airway epithelial cells [69–71]. These airway-epithelium injuries decrease the protective capacity of the epithelium against inhaled oxidants and further enhance inflammation. Therefore, oxidative stress causes airway inflammation, which gives rise to a further oxidative burden, thereby forming a vicious cycle.

ROS are known to increase mucus hypersecretion through neutrophilic activity [72] and contribute to the pathogenesis of chronic bronchitis. Increased neutrophil numbers in the airways can be detected in patients with acute COPD exacerbations. Epithelial growth factor receptors (EGFRs) play an important role in mucus hyperplasia and secretion. EGFRs can be activated by neutrophilic inflammation through neutrophil elastase secretion and might contribute to mucus hypersecretion [73].

Further, oxidative stress can increase the risk of bronchial hyperresponsiveness (BHR). Previous reports state that half of population of patients with COPD are diagnosed with BHR [74, 75]. ROS can induce tachykinin release from the capsaicin-sensitive sensory that act on the neurokinin receptors in the airways to induce BHR [76]. Also, lipid-peroxidation products are reactive molecules that can cause smooth-muscle contraction and subsequently contribute to BHR [77]. BHR is a strong predictor of the progression of airway obstruction in early COPD patients who continue smoking.

Damage caused to DNA by oxidative stress can be repaired by efficient DNA-repair machinery. However, failure to repair double-stranded DNA breaks leads to an increased risk of developing lung cancer in COPD patients [78]. ROS also result in protein carbonylation that may lead to the generation of circulating autoantibodies that cause damage to alveolar cells, particularly in severe COPD [79].

### Therapy strategies

Recent therapeutic choices for COPD include anticholinergics, β2-agonists, and inhaled corticosteroids, all of which ameliorate symptoms rather than curing the disease [80, 81]. There are no clinically available treatments that prevent COPD progression. Currently, several endogenous non-enzymatic and enzymatic antioxidants antioxidants are widely used to combat oxidative stress in the lungs. The commonly used nonenzymatic antioxidants include glutathione [82], ascorbic acid [83], uric acid [84], α-tocopherol [85], and proteins used to prevent the Fenton and Haber–Weiss reactions [86]. The enzymatic antioxidants mainly include catalase, SOD isomers, and GSH-associated enzymes [87–89]. Although oxidative stress is an excellent target, these agents are not very effective. The most common obstruction faced by conventional antioxidant drugs involves airway immune response, mucous hypersecretion, inflammation, and low bioavailability. Overall, studies of anti-oxidants in COPD have been disappointing. There remains a considerable need to develop novel drug therapies for patients with COPD. However, the introduction of a novel DDS can help improve the pharmacokinetic behaviour of drug molecules inside a biological entity, such as the rapid absorption of the nano/mirco-drug owing to its high surface area. DDS can enable sustained release in the lung tissue, prolong drug circulation time, reduce dosing frequency, and improve patient compliance. Local DDS, such as inhaled medicines, might be an alternative that could decrease the incidence of side effects associated with high drug-serum concentrations.

### Novel drug delivery systems

**Fig. 3:**
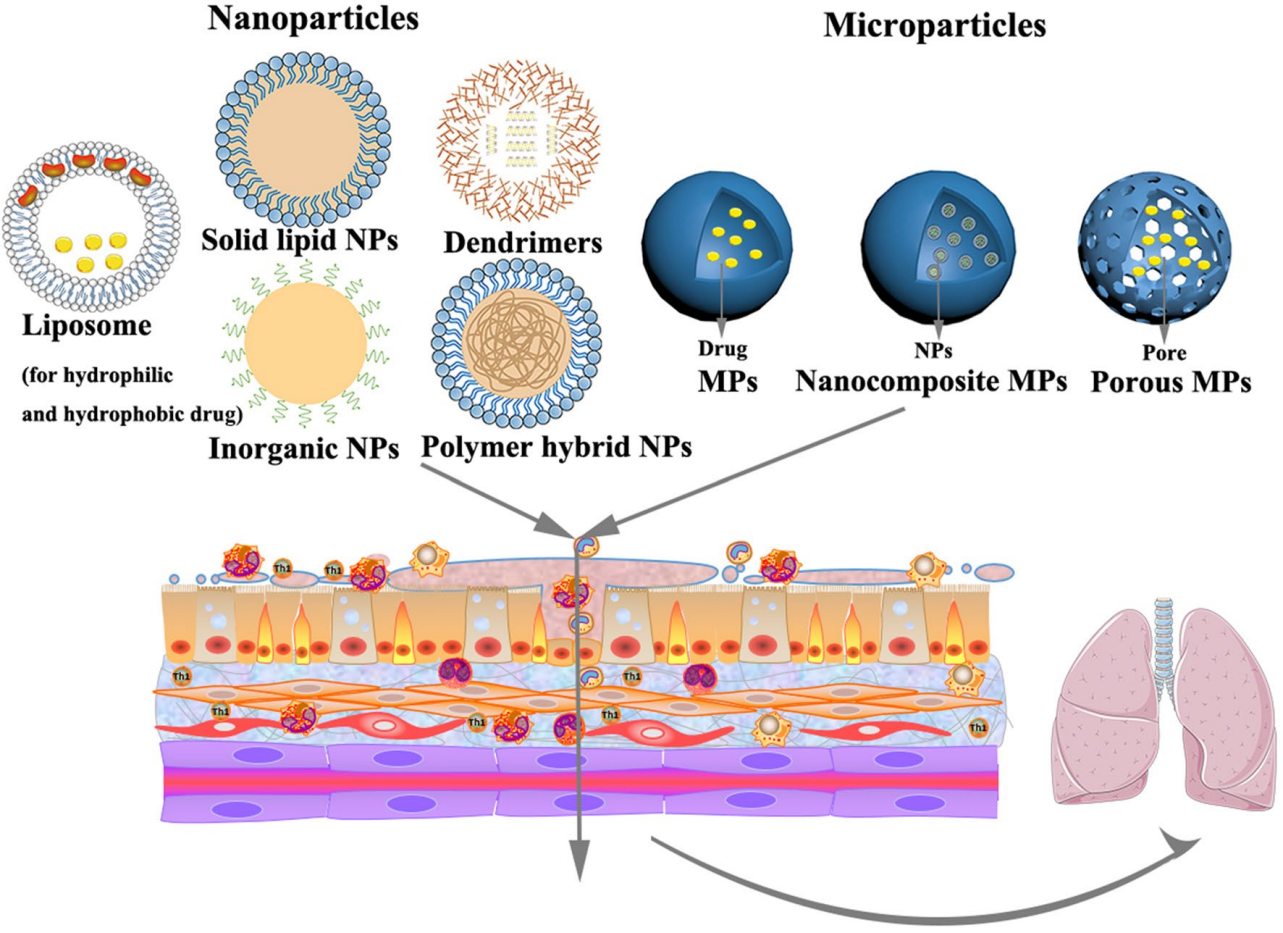
Representative nanoparticles and microparticles used for delivering antioxidants in COPD

Novel DDS have gained increasing attention in the treatment of COPD owing to their advantages of targeted deposition, sustained release, biodegradation, reduced dosing frequency and well controlled size and surface charge. Owing to their small size, high surface-volume ratio, high stability, and unique physicochemical properties, nano/microcarriers can be utilized to incorporate hydrophilic and hydrophobic drugs as well as other biologics. These unique characteristics make nano/mircocarriers a promising platform for improving the solubility, dissolution, and bioavailability of therapeutics. Novel DDS include liposomes, polymeric nanoparticles, solid lipid nanoparticles, polymeric micelles, dendrimers, nanoemulsion, nanosuspension, and microspheres, and microparticles (Fig. 3). However, the application of DDS in COPD is still in its early stages. The progress in designing novel DDS against oxidative stress, oxidative-stress triggered inflammation, and challenges in translating technology into the market are discussed in the following sections.

### Liposomes

Liposomes are among the most established drug delivery platforms owing to their flexibility, biocompatibility, biodegradability, and nonimmunogenicity. Liposomes constructed using amphiphilic phospholipids have a spherical lipid structure containing an aqueous core [90]. The hydrophilic (aqueous core) and hydrophobic shell (spherical lipid structure) enable the transport of hydrophilic, hydrophobic, and amphiphilic molecules and make liposomes convenient for use in combination therapy, which is crucial for COPD treatment [91]. Many lipids commonly used to prepare liposomes have good compatibility with the lung tissue and can facilitate the intracellular delivery of several therapeutic agents via fusion with the plasma membrane lipids, receptor-mediated endocytosis, and phagocytosis owing to the diversity of the amphiphilic lipid molecules [92]. Liposomes are used in transporting hydrophilic, hydrophobic, and amphiphilic antioxidants as well as antioxidant enzymes to different organs and tissues for the treatment of oxidative stress-induced damage (Table 2).

**Table 2:** Relevant studies focusing on liposomes for oxidative stress

Nanocarrier composition	Drug	Method of preparation	Size	Route of administration	Mode of action	Ref
DPPC, cholesterol and stearylamine	CAT or SOD	Reverse-phase evaporation	–	Intravenous	Decrease lipid peroxidation products (malondialdehyde, conjugated dienes, lipid hydroperoxides)	[93]
DPPC	a-Tocopherol	Reverse-phase evaporation	320 ± 40 nm	Intratracheal	Reduce myeloperoxidase activity and reverse of phorbol myristate acetate-induced changes in lung edema, lipid peroxidation, enzyme and alkaline phosphatase activities	[94]
DPPC and cholesterol	Cu, Zn SOD and CAT	Reverse-phase evaporation	200 nm	Intratracheal, instillation	Increase antioxidant activity of alveolar type II cell, increase lung antioxidant enzyme levels	[95, 96]
DPPC	SOD and/or CAT	Reverse-phase evaporation	0.1–0.4 μm	Intratracheal	Prevent the chronic vascular and parenchymal damage due to oxygen toxicity	[97]
DPPC	NAC	Reverse-phase evaporation	-	Intratracheal	Increase pulmonary glutathione	[98]
DPPC	NAC, glutathione, a-tocopherol	Thin-film hydration method	100 nm	Intratracheal instillation	Reduce CINC-1, IL-1β, and TNF-α	[99]
Phospholipid and cholesterol	NAC	Reverse phase evaporation and spray drying	∼100 nm	Inhalation	Against TBARS production	[100]
DPPC	a-Tocopherol	Solvent evaporation method	-	Intraperitoneal, injection	Reduce acute inflammatory, cell influx and suppress collagen formation in lung tissue	[101]
Chitosan, hyaluronan, and phospholipids	Curcumin	Sonication, stirring	130 nm	A549 cells	Cell relative metabolic activity ≥ 80% after treated with hydrogen peroxide	[102]

DPPC: L-a-dipalmitoylphosphatidyl-choline; NAC: N-acetylcysteine; TBARS: thiobarbituric acid reactive species

Liposomes containing antioxidants are being studied for acute oxidant-related COPD. To date, liposomes have been widely used in the delivery of molecules with antioxidant properties, including the lipophilic antioxidants α-tocopherol; hydrophilic antioxidant glutathione, NAC, and the antioxidant enzymes SOD and CAT. However, the effectiveness of SOD and CAT is limited owing to their unfavourable physicochemical properties. Liposomal-entrapment markedly increases the activity of these enzymes [103]. Other antioxidant-loaded liposomes show promising results. For example, Hoesel et al. tested NAC-loaded liposomes and showed that they reduced lung permeability index in acute (4-h) CEES-induced injury by a 59%; Proinflammatory mediators in the bronchoalveolar lavage fluids from oxidant-expose lungs (CEES caused a 20-fold, fourfold, and 1.6-fold increase in CINC-1, IL-1β, and TNF-α, respectively) were reduced to baseline levels [99]. Curcumin-loaded liposomes are potential and safe delivery systems to prevent chronic inflammatory conditions and can be modified by coating their surface with chitosan or hyaluronan to protect both the phyto drug and vesicles and improve the local efficacy of curcumin. These liposomes improved the nebulisation performance and provided relatively good protection against oxidant-induced damage to the lungs. Particularly, a synergic effect of curcumin and hyaluronan was observed which resulted in a proliferative effect and a subsequent enhancement of the metabolic activity of cells [102]. To protect liposomes from degradation in vivo and prolong their activity, PEG was added to the surface of the liposomes, which generated liposomes with a hydrophilic ‘sterically stabilised’ surface. PEGylated liposomes have a lower affinity for macrophages in the mononuclear phagocyte system.

A major difficulty in translating liposome treatment from research to the clinic is delivery. Inhaled pharmacologic therapy is a cornerstone treatment for patients with COPD [104]. The administration of liposome could improve lung maintenance and lessen side effect. Thus, dry inhaled powder, Arikace and Pulmaquin, are currently in clinical-development phases [105]. Arikace is a dipalmitoyl-phosphatidylcholine and cholesterol liposome loaded with amikacin to treat *Pseudomonas aeruginosa* infections that affect the lung and ultimately lead to cystic fibrosis. Pulmaquin is a ciprofloxacin liposome used to the treat lung infections [106].

However, to date, inhaled liposome therapy has several limitations. Liposomes are associated with drug leakage owing to their relative instability during storage and nebulization and are also unstable during dehydration. For the product to be effective, it is essential to maintain the physical properties of liposomes after inhalation. Therefore, new approaches for preparing liposomes of increased stability are needed.

### Nanoparticles

Nanoparticles offer a new approach for the targeted delivery of drugs to the lungs [107]. They have been used mainly to target the intracellular cell adhesion molecule and platelet endothelial cell adhesion molecule receptors present on the endothelial cells of the pulmonary airway. Nanoparticles can be used as carriers to transport drugs, such as NADPH oxidase inhibitors, SOD and CAT, to prevent oxidative stress in the respiratory system [108]. Table 3 lists relevant studies focused on nanoparticle-based therapy for COPD.

**Table 3:** Relevant studies focusing on nanoparticle-based therapy for COPD

Type	Nanocarrier composition	Drug	Method of preparation	size	Route of administration	Mode of action	Ref
Solid lipid nanoparticle	Lipid and surfactant	Proanthocyanidins	Melt-emulsion method	243 nm	H441 cells	Reduce ROS production	[109]
	Lipid	Carvacrol	Fusion-emulsification method	78.72 nm	Inhalation	Minimize the inhalation injury by reducing malondialdehyde and minimize the histological change	[110]
Inorganic nanoparticles	Gold nanoparticles	Au	–	21 nm	Inhalation	AuNP can be used as nanocarrier (rapid binding to the alveolar epithelium)	[111]
	Ferrous and ferric chlorides	Antibody conjugates	Controlled precipitation approach	∼350 nm	Intravenous	Enable endothelial delivery of active ingredients and protected from proteolysis CAT and SOD	[106]
	Cerium oxide (IV) nanoparticles	SOD and CAT		2–3 nm	SOD enzymatic assay	Against ROS	[112]
	Al_2_O_3_ NPs	Al_2_O_3_	(Purchased from Plasmachem Gmb)		Inhalation	Al_2_O_3_ NPs exposure lead to suppression of PTPN6 and phosphorylation of STAT3. rescue of PTPN6 expression or application of a STAT3 inhibitor which protect lungs from inflammation and apoptosis	[113]
Biodegradable nanoparticles	Poly(ε-caprolactone)	Lipoic acid	Interfacial polymer deposition	191–349 nm	In vitro lipid peroxidation system	Protection against lipid peroxidation	[114]
	HPOX	HBA	Single emulsion method	~ 450 nm	RAW 264.7 cells and in vivo intranasally	inhibit NO production by suppressing iNOS expression in LPS-activated cells	[115]
	polyoxalate	HBA	Conventional single emulsion method	~ 500 nm	Intratracheally, injection	Scavenge H_2_O_2_, suppress the expression of iNOS, COX-2, (IL)-1β	[116]
	Poly(trolox ester)	trolox	Single-step emulsion technique	120–220 nm	U937 cells	Enzymatic degradation to release active antioxidants and suppress almost 50% of oxidative stress in the cells	[117]
Polymer nanoparticles	PHEA-PLA-PEG2000	FP	HPH (freeze drying)	161.3 ± 4.14.0 nm	Immortalized normal bronchial epithelial cell line	Improve drug permeation through the mucus layer, reduce the survivin expression	[118]
	PEG-DSPE	Budesonide	HPH (freeze drying)	∼550 nm	Inhalation	–	[119]
	PVP; PVA or dextran	Curcumin	Solvent and antisolvent precipitation method	30 nm	Inhalation	Inhibit LPS-induced inflammation in alveolar macrophages in a time dependent manner	[120, 121]
	PGA-co-PDL, cationic lipid DOTAP	microRNAs	Single emulsion solvent evaporation method	244.8 ± 4.40 nm	Human alveolar adenocarcinoma A549 cells	Reduce IRAK1 expression and dampen IL-8 promoter reporter output	[122, 123]
	PLGA, calcium phosphate, chitosan or PEI	siRNA, pDNA, FITC-BSA	Modified the rapid precipitation method (freeze drying)	Below 200 nm	HeLa cells	Increase the encapsulated siRNA or DNA and help them across the cell membrane	[124, 125]
	PLGA, calcium phosphate, polyethylenimine	siRNA	Modified the rapid precipitation method (freeze drying)	~ 145 nm	Nasal instillation	Regulate the expression of IFN-γ, CCL-2 and IP-10 to achieve a decreased inflammation of the lungs	[124, 125]
Dendrimers	PEGylated polylysine dendrimers	–	[126]	11–78 kDa	Pulmonary instillation	Control delivery of medications to lungs by modified with variously sized PEG groups in particle surface	[127]
	PAMAM dendrimers (PEGylated or not)	–	–	5.1–9.9 nm	Pulmonary delivery pharyngeal aspiration (P.A.) technique	Enhance dendrimer reaching the endothelial cells and systemic circulation. P.A. administration promotes the passive targeting of dendrimers to lymph nodes	[128]
	TEE modified PAMAM dendrimers	siRNA	Vortex	257 nm	Inhalation	Target lung alveolar epithelial A549 cells and silence genes	[129]
	PAMAM dendrimer	TNF-α siRNA	Vortex	127–153 nm	RAW264.7 cells, intranasal in acute lung inflammation model	Gene silence (targeted TNF-α)	[130]
Polymer hybrid nanoparticles	PLGA and DOTAP	siRNA	DESE	Below 250 nm	H1299 cells	Gene silence (targeted TNF-α)	[131]
	PLGA and DOTAP	pHDAC2, MnPD	Modified solvent displacement method	~ 120 nm	A549 cells	Reduce ROS level and glucocorticoid resistance	[123]
Nanocrystals	Pluronic F68 or lecithin	Budesonide	Wet-milling technique	150–400 nm	–	Facilitate easier industrial use of nanocrystals	[132]
Multifunctional nanomaterials	Fibroin	Sulforaphane, CeNPs and PEI passivated CDs	Modified solvent displacement method	365 ± 20.2 nm	Cell evaluation	Against oxidative stress and imaging	[133]

DESE: double emulsion solvent evaporation method; HBA: p-Hydroxybenzyl alcohol; trolox: antioxidant and water-soluble analogue of Vitamin E; Al_2_O_3_ NPs: aluminum oxide nanoparticles; FP: fluticasone propionate; HPH: high-pressure homogenization; PLGA: poly(lactide-co-glycolide); HPOX: HBA-incorporated copolyoxalate; LPS: lipopolysaccharide; COX-2: cyclooxygenase-2; U937: Human leukemic monocyte lymphoma cells; PEG-DSPE: Polyethylene glycol and phosphatidylethanolamine; PVP: polyvinylpyrrolidone; PVA: polyvinyl alcohol; PGA-co-PDL: poly (glycerol adipate-co-ω-pentadecalactone; DOTAP: dioleoyltrimethy- lammoniumpropane; PLA: poly(lactic acid); pHDAC2: HDAC2-encoding plasmid DNA; MnPD: Mn-porphyrin dimer; CeNPs: cationic cerium oxide nanoparticles; CDs: carbon dots

#### Solid lipid nanoparticles

Solid lipid nanoparticles (SLNs) are biocompatible and markedly more stable than liposomes. Their production can be easily scaled up (high-pressure homogenisation and simple emulsification); and they are innocuous compared to polymeric nanoparticles. Castellani et al. prepared a SLNs based DDS using a melt-emulsion method to encapsulate proanthocyanidins and that showed it lowered oxidative stress by reducing ROS production. SLNs demonstrated non-significant toxicity against airway epithelial cells. The uptake and persistence of SLNs in airway epithelial cells in vitro were more extensive compared to free drugs and demonstrated prolonged residence time when administered in vivo. SLNs were taken up in a dose-dependent manner and persisted in cells up to 16 days. Further, SLNs were stable at 4 °C in double-distilled water for up to 2 months [109]. Carvalho et al. prepared SLNs containing carvacrol using a fusion-emulsification method. The polydispersity index (PDI) of the products was 0.126 ± 0.015 and the average size of SLNs was 78.72 ± 0.85 nm. SLNs containing carvacrol minimised the inhalation injury by significantly reducing malondialdehyde levels and minimized the histological changes [110].

However, SLNs have several limitations concerning their application, including unpredictable drug-release behaviour and the potential for gelation owing to the polymorphism of solid lipids [134, 135].

#### Inorganic nanoparticles

Inorganic nanoparticles are composed of noble metals such as gold and silver or inorganic materials such as calcium phosphate, carbon, silicon oxide, and iron oxide. Inorganic nanoparticles generally possess versatile properties suitable for pulmonary delivery including wide availability, rich functionality, good biocompatibility, and potential capability of targeted delivery. Inorganic nanoparticles have also been assessed as potential nanocarriers for COPD. For example, cationic metallic nanoparticles are being used for gene delivery because they can easily bind to anionic DNA/RNA [136]; gold nanoparticles can be used for targeted delivery to epithelial cells in COPD [111]. Hood et al. described antibody conjugates loaded with ferrous and ferric chloride antioxidant nanoparticles for endothelial targeting which could target the endothelial delivery of CAT and SOD. These inorganic nanoparticles protected from proteolysis CAT and SOD and enabled endothelial active delivery, which provided specific protective antioxidant and anti-inflammatory effects in animal models of acute inflammation and/or oxidative stress [106]. Gil et al. introduced inorganic cerium oxide (IV) nanoparticles conjugated with the antioxidative enzymes, SOD and CAT, to scavenge oxygen and nitrogen radicals. The results showed that SOD/CAT and the nanocarrier could complement each other and provide a synergetic antioxidant activity. Nonetheless, this approach needs to be explored further in in vivo studies [112]. Antioxidant enzyme nanocarrier formulations can be used to target endothelial cells to generate anti-inflammatory effects.

Inorganic nanoparticles exhibit intrinsic oxidant-generating properties. Some silver nanoparticles were found to induce oxidative stress in a dose- and time‐dependent manner, as indicated by the depletion of GSH and induction of ROS, SOD, and CAT [137, 138]. Avoiding the oxidation of nanoparticles is the most important aspect in the design of antioxidant inorganic nanoparticles.

#### Polymeric nanoparticles

Polymeric nanoparticles have an adaptable nature with physicochemical qualities that can change to accommodate different purposes. Different polymers can be used to reduce dosing frequency and target specific cells or organs, adjust the release pattern, and adjust the particle size and surface charge to escape alveolar macrophage clearance or facilitate transepithelial transport. The commonly used polymeric materials include natural polymers such as albumin, chitosan, gelatin and HA; synthetic polymers such as PEG, PVA, PLA; and copolymers, such as PLGA. Moreover, it has been recognised that polymers usually do not induce a strong immune response [139]. Mohamed et al. used PGA-co-PDL and DOTAP to prepare nanoparticles loaded with miR146a to reduce the expression of the IRAK1 target gene. miR146a delivered as miR-146a-NPs reduced the IRAK1 expression to 40% and dampened the IL-8 promoter-reporter output. These results demonstrate the potential of PGA-co-PDL NPs as a delivery system for miR-146a to treat COPD [122]. By transforming polymer nanoparticles into microparticles via spray drying, researchers have demonstrated the potential of transforming polymer nanoparticles into industrial preparations.

The development of a biodegradable NP-based DDS further optimize the application of polymeric nanoparticles as they can effectively avoid the accumulation of polymeric materials following repeated dosing. CS, HA, and PLGA have been frequently reported to have promising prospects. Biodegradable hydroxybenzyl alcohol-incorporated polyoxalate (HPOX) is a novel biodegradable nanoparticle material used to combat oxidative stress in airway inflammatory diseases. During the degradation of peroxalate ester linkages, HPOX leads to the releases of HBA in vitro. HPOX administered intranasally induces a significantly reduced expression of iNOS and attenuates allergic inflammation. No cytotoxicity was observed with cells treated with HPOX nanoparticles less than 100 μg [115]. Biodegradable nanoparticles includes PLGA and PEG which enable the biodegradation of nanoparticles after administration [140, 141]. Further advancements have made by using biodegradable polymeric hydrogel for pharmacological applications. For example, sodium alginate (ALG)-Fe has tissue-mimicking, mechanical properties and biocompatibility [142].

Biodegradability and biopersistence of nanoparticles can influence nanoparticle-cell interactions, which may have direct effects on cellular or organelle oxidative status, or indirect effects through inflammatory processes and enzymatic detoxification pathways [143, 144]. Although the introduction of biodegradation provides opportunities to overcome airways defence and avoid the accumulation of polymeric materials, it has potential safety issues in other aspects. For example, the complete degradation of PLGA take months. The degradation products, lactic acid and glycolic acid, can accumulate and cause changes in a disturbance in the microenvironmental pH [145]. Therefore, it is highly recommended to determine the polymer degradation rate and long-term safety before inhaled polymeric DDS are used clinically.

#### Dendrimers

Dendrimers represent a promising class of nanocarriers for drug delivery to the lungs. The commonly used polymeric materials include natural polymers, such as poly(amidoamine) (PAMAM), ploy(_L_-lysine) (PLL), polyamides, polyesters (PGLSA-OH), polypropylenimine (PPI), ploy (2,2-bis(hydroxyl methyl) propionic acid), and ployethers [146]. PEGylated dendrimers have been used to reduce toxicity, improve pharmacokinetic profiles, and improve aqueous solubility. The degree of PEGylation can significantly effect dendrimer splitting and retention. Ryan et al. examined the potential utility of PEGylated poly(lysine) dendrimers as pulmonary delivery agents. Larger PEGylated dendrimers may be retained in the lungs and enable controlled drug delivery [127]. Zhong et al. investigated the systemic and lung cellular biodistribution of generation 3, PAMAM dendrimers (G3NH2) [128]. PAMAM dendrimers prolonged systemic circulation and accumulated effectively in the lungs through passive circulation. PAMAM dendrimers also show potential in delivering biomacromelucles, such as siRNA [129, 130].

#### Lipid-polymer hybrid nanoparticles

Lipid-polymer hybrid nanoparticles (LPNs) exhibit complementary characteristics of both polymeric nanoparticles and liposomes, particularly in based on their physical stability and biocompatibility. LPNs have been demonstrated to exhibit superior efficacy for in vivo cellular delivery. Thanki et al. developed lipidoid-modified (lipid like materials) LPNs consisting of PLGA and lipidoid. Lipidoid-modified LPNs showed strong gene silencing effects in the human non-small lung carcinoma cell line H1299 [131]. Lipidoid-modified LPNs offer promising prospects for the efficient and safe intracellular delivery of siRNA for COPD [147]. Lipidoid consist of an alkylated tetraamine backbone. Different analogues have been obtained, depending on the degree of alkylation. Compared to the commonly used cationic lipids such as DOTAP, lipidoids contain multiple secondary and tertiary amines. Therefore, they are more efficient in interacting with anionic siRNA molecules without significantly increasing the net charge of the LPNs. Another LPNs are a core–shell type LPNs composed of a PLGA core encapsulating a potent antioxidant Mn-porphyrin dimer (MnPD) and a cationic lipid (DOTAP) shell that binds the pHDAC2. The transfection of pHDAC2 combined with the elimination of ROS by MnPD exhibited a significant enhancement of intracellular HDAC2 expression levels, suggesting that the multi-antioxidative activity of MnPD plays a crucial role in the expression of HDAC2. The nanocarriers of LPNs are suitable for delivering drugs for COPD [123]. Efficient and simple large-scale production of nanoparticles has already been developed; however, attempts continue to scale-up the production of LPNs and simplify their preparation.

#### Multifunctional nanoparticles

Multifunctional nanomaterials integrating therapeutic and imaging modalities have opened a new era in the present therapeutic strategies, namely, theranostics. Two-dimensional (2D) nanosheets, a type of brand-new nanomaterial, which could integrates multiple functionalities of various materials to obtain an ‘all-in-one’ platform is a potential starting point for translational research [148]. Passi et al. designed a multifunctional silk fibroin-based carrier for the delivery of antioxidant and imaging agents. A one-step desolvation method was used to prepare sulforaphane (antioxidant drug)-loaded silk fibroin nanoparticles (SFSNPs). These anionic SFSNPs were further coupled with CeNPs and PEI-passivated carbon dots (CDs) to form self-assembled CeNP-CD@SFSNPs nanocomposites. The synthesised CeNP-CD@SFSNPs nanocompositescould could efficiently reduce ROS levels while simultaneously enabling imaging of the cells [133]. Such multifunctional nanocomposites could be potential candidates for the delivery of drugs to prevent oxidative stress, simultaneously, enabling the detection of pathological conditions in patients.

Nanotechnology provides a new dimension to the targeted drug-delivery approach, with multiple benefits in COPD. However, nanomaterials for transporting therapeutics need to be assessed for the potential health hazards, especially since different nanoparticles have been demonstrated to induce toxicity related to their nanometre size, opening a the new field of nanotoxicology [149]. Nanoparticles also generate and contribute to oxidative stress via direct and indirect cellular interactions. Nanoparticle interaction and damage to internal cellular structures can lead to oxidation, which further exacerbate the severity of oxidative stress. Additionally, nanoparticles may indirectly interact with cells to alter ROS production and emission through modified cellular phagocytic activity and oxidative burst. Liu et al. evaluated the oxidative stress caused by silica nanoparticles (SiO_2_ NPs). The fluorescent probe, DCFH-DA, was used to detect ROS by measuring the fluorescence intensity. Interestingly, the fluorescence was about 1.7 times stronger in cells exposed to SiO_2_ NPs than in those exposed to microsized SiO_2_ particles. The finding suggested that the oxidative damage in cells exposed to SiO_2_ NPs was more serious than that in A549 cells exposed to microsized SiO_2_ [150]. This is because SiO_2_ NPs have a smaller size and larger surface area, which can more efficiently interact with cellular or subcellular structures. However, there is an overwhelming amount of toxicity data derived from environmental health studies, which shows that nanoparticles can induce oxidative stress. Whether the results apply to nanoparticle therapeutics remains controversial. The induced toxicity and oxidative stress of nanoparticles must be considered when designing nanoparticles for the treatment of oxidative stress-induced COPD.

Nanoparticle technology offer effective approach for drug delivery in COPD. However, several issues remain to be resolved prior to its use in a clinical setting. To date, no clinical studies using nanoparticles in the treatment of oxidative stress have been registered at the ‘clinical trials.gov’ database. Inhaled pharmacologic therapy is a cornerstone of treatment for patients with COPD, which have a wide range of advantages such as requirement of low concentrations of drug to reach therapeutic efficacy, surpassing first pass metabolism and a very low incidence of side effects. From an aerodynamic viewpoint, nanoparticles cannot be directly used for inhalation because they are not in the optimal size range for inhalation (ideal median mass aerodynamic diameter (MMAD) for deposition in the small airways and alveoli of the lungs should be 1 to 5 µm). Individual nanoparticles used for inhalation are prone to exhalation instead of deposition in deep lung owing to the low inertia of nanoparticles. Additionally, nanoparticles have larger surfaces, which results in an increase their free energy and increased the interaction between particles. The high degree of interaction between nanoparticles leads to particle agglomeration or drug-crystal growth [151, 152]. To address these problems, nanoparticles should be entrapped within microparticles. The solid matrix can prevent the interaction between nanoparticles, limit their mobility, and increase their long-term stability. More importantly, it can improve aerosolization properties and improve pulmonary administration.

### Microparticles

Microparticles are rapidly gaining popularity owing to their controlled-release properties, high drug loading and entrapment efficiencies, particle size, therapeutic benefits, and compatibility, among other advantages. In particularly, the delivery of microparticles via inhalation is becoming an area of remarkable interest for researchers in the field of respiratory medicine [153].

**Fig. 4:**
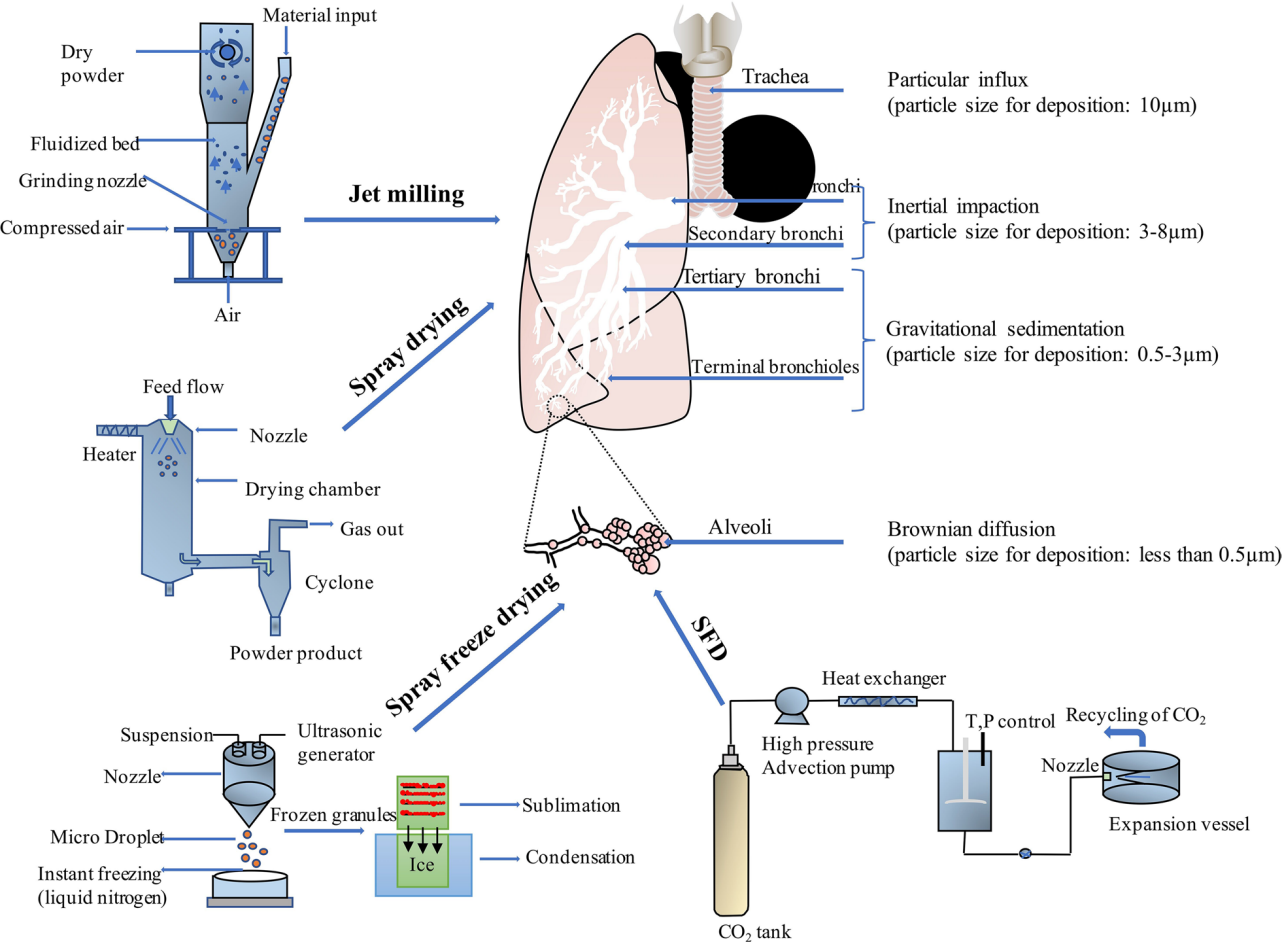
Manufacturing methods, jet milling, spray drying, spray freeze drying and SFD, of preparing microparticles and their deposition mechanism

Microparticle inhalers have become increasingly attractive for the pulmonary delivery of locally and systemically effective medications. Dry powders show appropriate morphologies and suitable aerosol properties for inhalation drug delivery. Manufacturing methods including jet milling, spray drying, spray freeze drying and supercritical fluid (SFD) technology are capable of generating particles ranging from 1 to 5 um in size and have been used to prepare dry powders with desired properties. Depending on the particle size, microparticles are deposited in the lungs via the three mechanisms, namely, gravitational sedimentation, inertial impaction, and Brownian diffusion (Fig. 4). As particles with larger diameters (usually > 10 μm) are suitable for deposition in the oropharynx and those with smaller diameters (usually < 0.5 μm) are easily exhaled, microparticles in the range of 1 to 5 μm are optimal for achieving effective pulmonary deposition. This phenomenon is referred to as an impaction; particles with aerodynamic diameters between approximately 1 and 5 μm are deposited slowly in the narrow airways and bronchioles by sedimentation [154]. In addition to particle size, other properties influence aerosol performance, namely, particle density, shape, crystallinity and polymorphism, inter-particulate forces and surface roughness [155].

Relevant studies focusing on microparticles for oxidative stress are summarized in Table 4.

**Table 4:** Relevant studies focusing on microparticles for oxidative stress inhabition

Type	Drug	Key excipient	Method of preparation	Size	Mode of action	Ref
Microscale dry powder	A 1,2,4-triazole motif and quinolinone	Lactose	Jet milling	Less than 2 μm	Bifunctional MABAs	[156]
	FP, MF and SX	–	(1) Jet milling (2) Wet polishing	(1) 2.18–2.53 μm (2) 0.87–1.49 μm	Corticosteroids and long-acting β2-agonists	[157]
	SBS or BD	–	(1) Jet milling (2) Spray-drying	(1) 0.59–0.65 μm (2) 0.63–0.69 μm	Short acting β agonist, synthetic glucocorticoid	[158]
	dimethyl fumarate	Mannitol	Co-spray dried	0.56–1.08 μm	Nrf2 activator drug to treat pulmonary inflammation	[159]
	naringin	Ethanol –water (50:50 v/v)	Co-spray dried	3.5 µm	Free-radical scavenger drug	[160]
	Indacaterol and glycopyrronium	–	–	–	Decrease IL-8, IL-10, TNF-α, MMP-9, PON1, increase TIMP-1 and MDA	[161]
	BD and RES	Ethanol –water (80:20 v/v)	Co-spray dried	1.0 µm	DECREASE the levels of TNF-α and IL-6 in LPS induce alveolar macrophages	[162]
	Resveratrol	Ethanol –water (50:50 v/v)	Spray-drying	3.86 µm	Scavenge activity of more than 50% of DPPH free radicals	[163]
	Resveratrol	Ethanol–water (50:50 v/v)	Spray-drying	3.9 μm	The expression of IL-8 from Calu-3 induced with TNF-α, TGF-β1 and LPS were significantly reduced	[164]
	BD	HA	Spray-drying	3.12–5.35 μm	Glucocorticoid	[165]
	Sodium ascorbyl phosphate	HA	co-spray dried	3.4 µm	ANTI-inflammatory, antioxidant, and wound healing properties	[166, 167]
Porous microparticles	Anthocyanin	PLGA microparticles, HA, β-cyclodextrin (porogen);	W1/O/W2 multi-emulsions freeze drying	5 ~ 10 μm	Sustain ATH release characteristics and protract antioxidant activity for DPPH radicals	[168, 169]
	BD	PLGA and PVP	modified single emulsion (O/W) solvent evaporation freeze drying	6 μm	–	[170]
	DEX	PVAX	Double emulsion method freeze drying	13 μm	Scavenge hydrogen peroxide, diminish oxidative stress	[114]
Mucoadhesive solid lipid microparticles	FP	Alginate, chitosan and lipid	Ethanolic precipitation technique (freeze drying)	1 ~ 5 μm	ERK1/2 pathway activation	[171]
	SX	Sodium alginate, Pluronic F68 and lipid	HPH (freeze drying)	3.3 μm	Long-acting β2 agonist	[172]
NCMPs	NAC	(1) Phospholipidand, cholesterol; (2) Lactose	(1) Reverse phase evaporation method (2) Spray drying	7.2 μm	Against TBARS production	[100]
	MicroRNA	l-Leucine and mannitol	(1) Oil in water (o/w) single emulsion method; (2) Spray-drying	4.20 ~ 6.03 µm	Genes silence of IRAK1 and TRAF6	[122]
	siRNA	(1) Lipidoid, PLGA (2) mannitol or trehalose	(1) DESE (2) Spray-drying	3.3 µm	Dispersed microembedded LPNs had preserved physicochemical characteristics as well as in vitro siRNA release profile and gene silencing	[173]
	siRNA	(1) dendrimer (2) mannitol, trehalose, inulin	(1) bulk mixing and microfluidics-based mixing (2) spray-drying	4.8 ~ 5.6 μm	The gene silencing efficiency of the nanocomplexes is preserved upon spray drying	[174]
Nanocomposite microparticles (NCMPs)	Curcumin	(1) PLGA; PEG-g-Cs copolymer or Cs	(1) Modified single emulsion − solvent evaporation method; (2) Spray-drying	3.1–3.9 μm	Microparticles have minimal propensity to induce TNF-α release which showed much delayed and reduced macrophage uptake	[175]
	BD	(1) TPGS (2) Leucine or albumin	(1) High-energy wet media milling; (2) Spray drying	4.39 ~ 5.30 μm	Anti-inflammatory activity	[176]
	Apigenin	(1) BSA (2) lactose and l-leucine	(1) Modified nanoparticle albumin-bound technology (2) Spray drying	2.47 μm	Antioxidant activity of drug is preserved and enhanced by the BSA;, scavenge the DPPH free radial	[177]
Clinical study	Ribavirin –PRINT –CFI	35% ribavirin with 55% trehalose and 10% trileucine	Non-wetting Templates (PRINT) technology	1 μm	Against the key respiratory viruses that can cause acute exacerbations in COPD	[178]
	Ribavirin-97 PRINT-IP	1% PVA	Non-wetting Templates (PRINT) technology	1 μm		

(1) Refers to microparticle or nanoparticle material; (2) refers to matrix materials.

MABAs: muscarinic antagonist and β2 agonist properties; NCMPs: nanocomposite microparticles; Nrf2: dimethyl fumarate activator; MF: mometasone furoate; SX: salmeterol xinafoate; SBS: salbutamol sulphate; BD: budesonide; RES: resveratrol; HA: hyaluronic acid; PON1: Paraoxonase; DPPH: 2,2-diphenyl-1-pikryl-hydrazyl; MDA: Malonyl dialdehyde; HSPB5: Alpha B-crystallin; DEX: Dexamethasone; PVAX: vanillyl alcohol-containing copolyoxalate; HPH: high-pressure homogenization; SX: Salmeterol Xinafoate; TPGS: D-α-tocopherol polyethylene glycol 1000 succinate; BSA: bovine serum albumin

#### Microscale powders

##### Microscale dry powder

**Fig. 5:**
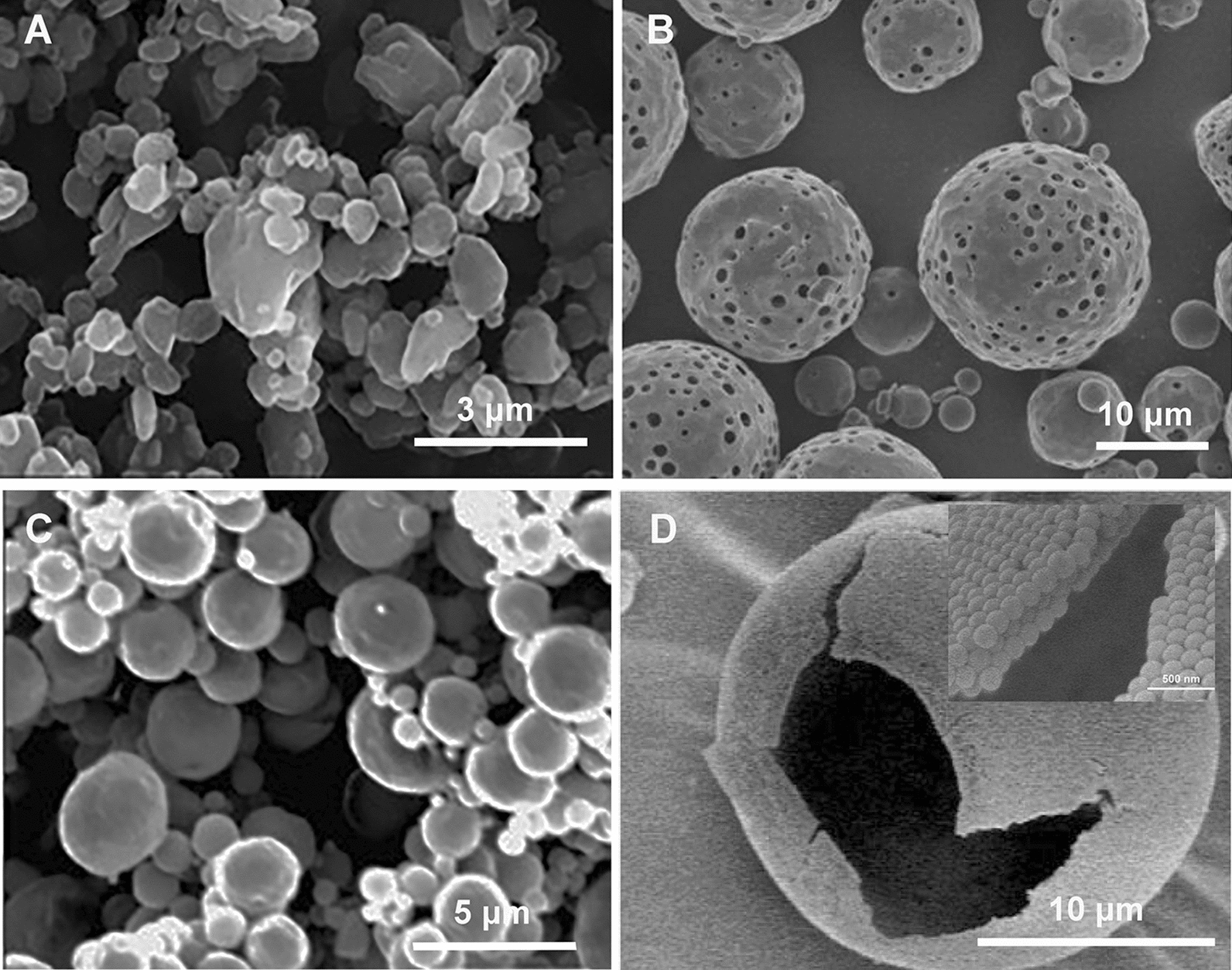
Morphology of **A** microscale dry powder [180]; **B** porous microparticles [170]; **C** matrix NCMPs [181]; **D** hollow NCMPs [182]

Advanced particle engineering design technology has been used to prepare inhalable microscale powders. The representative image of microscale dry powder can be seen from Fig. 5A. Muralidharan et al. reported the treatment of pulmonary inflammation by inhalation of microparticulate powders with dimethyl fumarate, an antioxidant Nrf2 activator. The spray-dried and co-spray-dried particles were prepared by advanced spray drying of an organic solution in a closed mode and employing D-mannitol as an aerosol performance enhancer. The dried powder had high drug loading with good in vitro aerosol dispersion performance. Spray-dried mannitol has a MMAD of 0.56 μm and a fine particle fraction (FPF) of 49%. Using in vitro studies of lung-deposition model, studies reported that aerosol particles can reach the lower pulmonary tract and treat inflammation during COPD [159]. A similar co-spray dried powder was described by Trotta et al. In their study, inhalable microparticles containing BD and RES were prepared and characterised to develop a multi-drug inhalable formulation with antioxidant and anti-inflammatory activities for the treatment of COPD [162]. The preparation of microscale powder is simple and easy to scale-up; however, drug loading and short duration of action are commonly encountered challenges [179].

##### Porous microparticles

The short systemic circulation time of microparticles remains a challenge owing to the rather short duration of action [6, 7]. Aerosolization of particles is an important property for inhalation and depends on particle–particle and particle–wall interactions. Microparticles with pores have a lesser tendency to aggregate, exhibit decreased phagocytosis by alveolar macrophages, and have higher aerosolization efficiency (Fig. 5B). The most widely exploited method for preparing porous microparticles is the multiple emulsion (w/o/w) approach with a porosity-inducing agent (such as β-cyclodextrin). Zhang et al. developed BD-loaded large porous microparticles (LPPs) for inhalation. The optimised formulation showed desirable aerodynamic behaviour to allow for drug delivery to the lungs [170]. Porous microparticles can also be used to load other types of antioxidative drugs for COPD. Many new materials have also been used for the preparation of porous particles because of their excellent biocompatibility, biodegradable and other properties. Jeong et al. developed a new family of biodegradable polymers, vanillyl alcohol-containing copolyoxalate (PVAX), which could scavenge hydrogen peroxide and exert potent antioxidant and anti-inflammatory effects. PVAX microparticles reduced oxidative stress and suppressed the expression of pro-inflammatory TNF-α and iNOS. Porous PVAX microparticles with encapsulated dexamethasone show great potential as a therapeutic system to treat airways inflammatory diseases [114]. However, PVAX microparticles have a large diameter (13 μm), which can negatively influence the deposition into the deep lungs if administrated by inhalation. Porosity\makes large-scale manufacturing difficult, and the requirement of particle flow-ability increases the difficulty in designing microparticles. Therefore, more innovation is needed to generate new pharmaceuticals for this market.

##### Mucoadhesive microparticles

To prolong drug action in the lungs, mucoadhesive polymers have been explored for COPD. Mucoadhesive polymers have structures and functional groups with high affinity for mucosal surfaces. For example, amino groups in chitosan undergo electrostatic interactions with the anionic groups of the mucus, which increases the mucoadhesiveness of microparticles. Anionic alginate can form hydrogen bonds with the mucin layer of the mucosa [183]. Solid lipid microparticles (SLMs) have a strong ability to form hydrogen bonds with the mucin layer of the mucosa to control drug release. The therapeutic approach using FP-loaded microparticles offers a remarkable potential for the treatment of COPD. Amore et al. described alginate- and chitosan-based mucoadhesive SLMs for the effective delivery of fluticasone propionate to treat COPD. SLMs have a useful dimensions (MMAD is 3.5–4.0 μm) for pulmonary release of FP to the secondary bronchi [171]. Liu et al. prepared budesonide mucoadhesive microparticles containing hyaluronic acid by spray drying. The MMAD of microparticles was 3.12–5.35 μm, which was in the inhalable range; however, the FPF was 35.6% which had to be further improved. The study showed that budesonide loaded in the mucoadhesive microparticles exhibited a significantly prolonged T_max_, which substantially delayed absorption and prolonged the retention of budesonide. This resulted in an increased bioavailability in an animal model owing to the mucoadhesive ability of hyaluronic acid [165]. Despite the success of mucoadhesive microparticles for the pulmonary delivery of antioxidant drugs, the release pattern and drug encapsulation efficiency should be considered when using microparticles. The redispersion of mucoadhesive microparticles is not feasible and should be evaluated before administration.

#### Nanocomposite microparticles

##### Nanocomposite microparticles in research

**Fig. 6:**
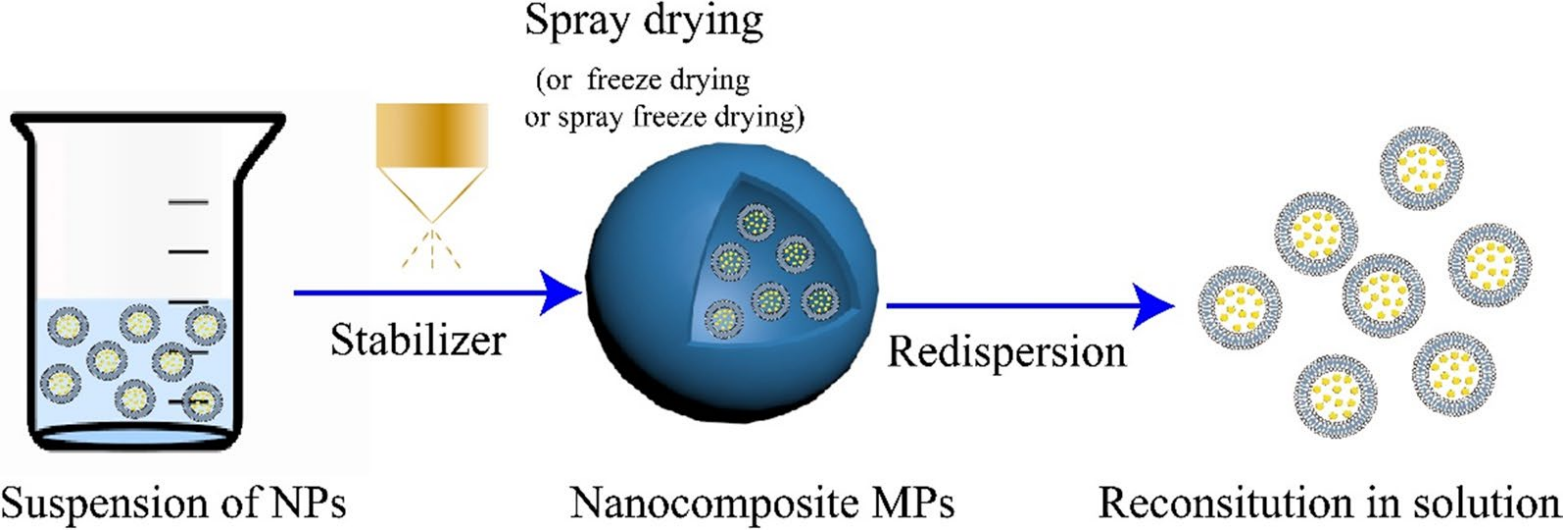
Preparation of nanocomposite microparticle and their reconstitution

Studies combining the merits of nanoparticles with the delivery convenience have been conducted by spray drying nanoparticles with or without additional excipients; the particles obtained using this approach are termed nanocomposite microparticles (NCMPs), which have the advantage of both nanoparticles and microparticles. The represent image of NCMPs can be seen from Fig. 5C, D. Under physiological conditions, NCMPs disassociate into the original nanoparticles and maintain the properties of the nanocarriers (nano complexes or nanoparticles), including their drug release and delivery advantages (Fig. 6). NCMPs exhibit better in vitro antioxidant activity than microparticles containing the non-encapsulated drug, which makes it a good candidate for the treatment of oxidative stress [100, 184]. Most of the NCMPs were synthesised using non-water-soluble or biodegradable polymers to atomise the aqueous suspension. Such microparticles can maintain the structure of nanoparticles after the drying process and translate into inhalable microparticle powder, which is more suitable for the treatment of COPD. NAC was efficiently encapsulated in liposomes produced by the reverse-phase evaporation method. Powders containing these liposomes presented suitable properties for pulmonary administration. A MMAD of 7 μm and respirable fraction above 30%, maintained or increased antioxidant activity after the drying process and recovered nano sized liposomes after aqueous redispersion. NAC dry powder possessed a higher antioxidant activity than the non-encapsulated drug in solution or dry powder containing the non-encapsulated NAC [100]. Mohamed et al. prepared NCMPs of microRNA (miR-146a) containing PGA-co-PDL nanoparticles for dry powder inhalation using l-leucine and mannitol as excipients. The microparticles maintained the bioactivity of miR-146a; however, after dispersion, the nanoparticles size increased, from 244.8 ± 4.40 nm to 409.7 ± 10.05 nm [122]. The microparticles showed a high FPF of 51.33% and MMAD ≤ 5 µm suggesting that deposition in the respirable region of the lungs would be possible. The selection of excipients and drying parameters is crucial for maintaining the least change in nanoparticles. In vitro drug release from microparticles depends on drug concentration in the nanoparticles, morphology of the final carrier, and the presence of pharmaceutical adjuvants [185, 186]. Generally, studies with NCMPs using drug-loaded nanoparticles aim to achieve a stable formulation with cellular uptake, long tissue retention times and sustained drug release. NCMPs are a promising approach for pharmaceutical nanoparticle processing and novel drug-delivery platforms.

To better control drug release, drug-loaded nanoparticles embedded within swellable microparticles represent another promising approach for drug delivery to the alveolar region where macrophage clearance occurs. Sherbiny and Smyth developed curcumin-loaded PLGA nanoparticles with chitosan-grafted-PEG or chitosan using spray drying. This process resulted in a series of respirable amphiphilic hydrogel microparticles derived by adjusting the PLGA content. The PLGA nanoparticles and the hydrogel microspheres showed sizes ranging from 221–243 nm and 3.1–3.9 μm, respectively. Hydrogel microparticles had desirable biodegradation rates, high drug loading (up to 97%), and good sustained release. Hydrogel microparticles had a minimal propensity to induce TNF-α release and showed delayed and reduced macrophage uptake [175]. These studies revealed that swellable microparticles could used as potential carriers for sustained pulmonary drug delivery. Current research on swellable microparticles for antioxidant delivery is still in its infancy. However, the key requirement of NCMPs is the spontaneous release of unaffected nanoparticles following pulmonary deposition. The in vitro and in vivo release patterns of swellable microparticles should be considered before further investigation.

##### Considerations in translating of NCMPs to the clinic

**Fig. 7:**
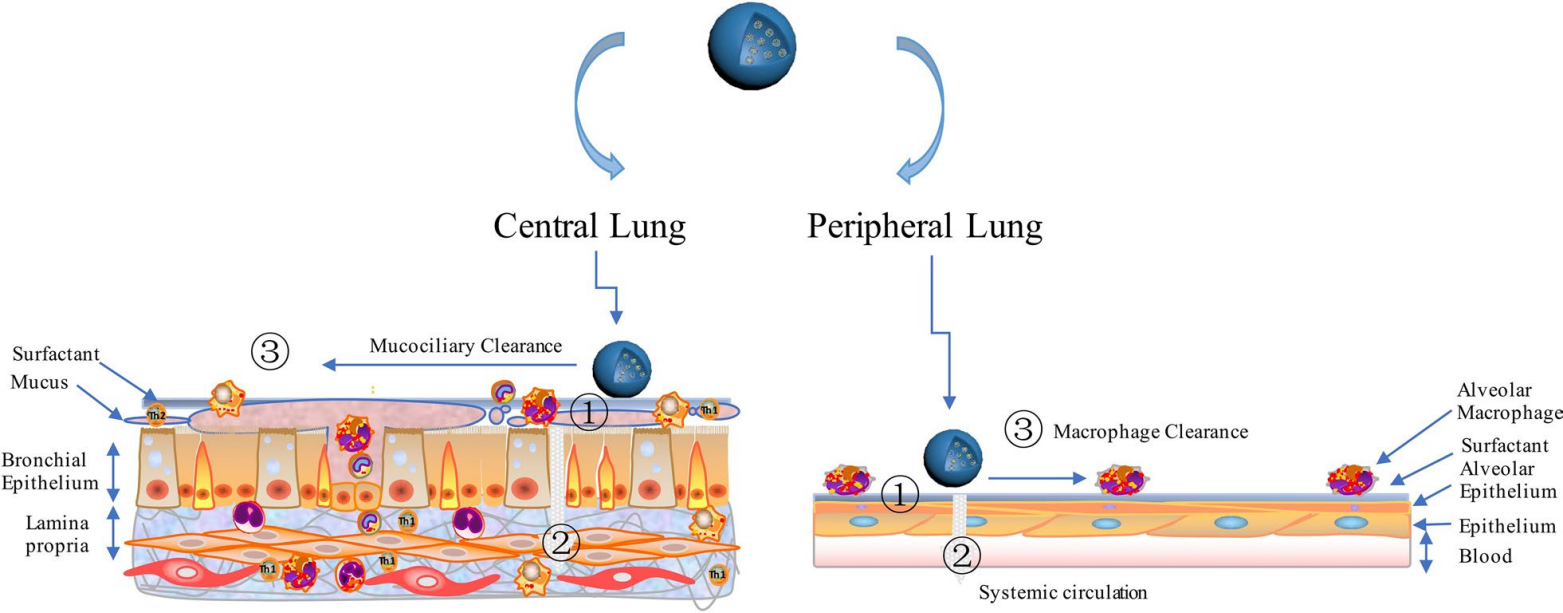
What happens to an aerosol drug after deposition in the lungs? (modified from [187])

The lung itself is an extremely complex structure, functioning as a significant barrier protect the respiratory system from pollutant particles and microorganisms. Many of the processes associated directly with respiratory functions present significant challenges for particle deposition, such as air humidification, temperature control in the thoracic and tracheobronchial regions, and gas exchange in the alveolar-interstitial region. Thus, particles must overcome lung-geometry barriers as well as lung physiology with high humidity (around 90%) during the respiratory phase, which can interfere with particle size and deposition. Particles must avoid mucociliary clearance and overcome the pulmonary surfactant layer that covers the alveolar epithelium in deep lungs. The fate of NCMPs includes the following three processes: First, the NCMPs need to translocate across the lung barrier and reach the bloodstream; NCMPs associate with the lining fluid or cells and are thus retained in the lungs for a long time; they are phagocytosed by macrophages. Overall, depending on the final particle destination, epithelial tight junctions, immunological cells, and lung lining fluids that represent additional barriers that must be overcome (Fig. 7).

#### Inhalable microparticles in clinical applications

Inhalable microparticles can be used to treat oxidative stress in COPD. Newly investigated inhalable microparticles are currently under clinical study. Dumont et al. used the Particle Replication In Non-wetting Templates (PRINT) technology to produce dry powder microparticles with uniform shape and size which is currently under Phase 1 study. Two new inhaled formulations of ribavirin (Ribavirin-PRIN-CFI and Ribavirin-PRINT-IP) were developed to achieve efficient delivery to the lung and minimise bystander exposure [178].

Therapies against oxidative stress and anti-inflammatory drugs are the mainstay treatments for COPD. However, frequent drug administration is associated with potential side effects. Thus, to ensure that drugs are administered less often, effective and safe sustained release drugs from of biodegradable microparticles is required for pulmonary delivery.

## Conclusion and future perspectives

Oxidative stress plays a major role in the pathogenesis and progression of COPD. It can cause airway inflammation, which further contributes to the oxidative burden. Although the mechanisms of airway inflammation and oxidative stress in COPD are apparent, the lack of an efficient DDS causes improper treatment that leads to chronic and fatal lung pathophysiology. There is an immediate need to develop novel DDS that can effectively deliver COPD therapeutics. The findings presented in this review suggest that nano/microtechnology-based therapeutics can be a more effective approach to treat oxidative stress. DDS have their advantages and disadvantages. Identification of the ideal nanoparticles properties is essential before making nanoparticles into microparticles to elicit the desired response and to acquire in-depth knowledge of the molecular and cellular mechanisms of oxidative stress in COPD. Liposomes are composed of biodegradable ingredients. They are easy to prepare, nonimmunogenic and show good aerosolization in solid form. However, there are also some requirements (mucus barrier, aerodynamic properties, clearance mechanism, absorption, and release properties) to be fulfil prior to clinical. SLNs (or nanostructured lipid carriers (NLC) improve drug retention and prolong drug release. For dendrimers and multifunctional nanoparticles, understanding the manufacturing and control requirements is very important. LPPs and NCMPs, such as LPNs, polymer nanoparticles, or biodegradable nanoparticle-loaded microparticles maybe the best option in COPD to prevent oxidative stress. However, despite some encouraging results, the clinical use of nano/micro-particles in the pulmonary field is still in the early stages. More research is needed to achieve efficient delivery of antioxidants to the lungs. Most importantly, potential lung toxicity (e.g., those caused by inorganic nanoparticles) and particle interaction with the immune systems could be risk factors that are far more severe than inflammation in COPD. Carefully designed preclinical studies on the safety and efficacy will help to move the nano/micro technology-based therapeutics closer to clinical evaluation in human subjects.

## Data Availability

Not applicable.

## Abbreviations

COPD: Chronic obstructive pulmonary disease
DDS: Drug delivery system
ROS: Reactive oxygen species
RNS: Reactive nitrogen species
iNOS: Inducible nitric oxide synthase isoenzymes
PM: Particulate matter
SLPI: Secretory leukocyte protease inhibitor
α1AT: Alpha-1-antitrypsin
IL: Interleukin
NF-κB: Nuclear factor kappa B
IL-8: Interleukin-8
GM-CSF: Granulocyte macrophage colony-stimulating factor
SOD: Superoxide dismutase
GPx: Glutathione peroxidase
iNOS: Inducible nitric oxide synthase
HDAC: Histone deacetylase
EGFRs: Epithelial growth factor receptors
BHR: Hyperresponsiveness
P13K: Phosphoinositide 3-kinase
TGF: Transforming growth factor
MMP: Matrix metalloproteinase
MUC5AC: Ne, neutrophil elastase
TNF: Tumor necrosis factor
CCL: CC-chemokine ligand
CCR: CC-chemokine receptor
CXCL: CXC-chemokine ligand
CXCR: CXC-chemokine receptor
T_H_1: T helper1 cells
T_C_1: Type 1 cytotoxic
NO: Nitric oxide
ONOO^−^: Peroxynitrite
DPPC: L-a-dipalmitoylphosphatidyl-choline
CAT: Catalase
NAC: N-acetylcysteine
TBARS: Thiobarbituric acid reactive species
HBA: P-Hydroxybenzyl alcohol
Trolox: Antioxidant and water-soluble analogue of Vitamin E
Al_2_O_3_ NPs: Aluminum oxide nanoparticles
FP: Fluticasone propionate
HPH: High-pressure homogenization
PLGA: Poly(lactide-co-glycolide)
PEG-DSPE: Polyethylene glycol and phosphatidylethanolamine
PVP: Polyvinylpyrrolidone
PVA: Polyvinyl alcohol
PGA-co-PDL: Poly (glycerol adipate-co-ω-pentadecalactone
DOTAP: Dioleoyltrimethy- lammoniumpropane
PLA: Poly(lactic acid)
PEG: Polyethyleneglycol
pHDAC2: HDAC2-encoding plasmid DNA
MnPD: Mn-porphyrin dimer
DESE: Double emulsion solvent evaporation method
HPOX: HBA-incorporated copolyoxalate
LPS: Lipopolysaccharide
COX-2: Cyclooxygenase-2
U937: Human leukemic monocyte lymphoma cells
CeNPs: Cationic cerium oxide nanoparticles
CDs: Carbon dots
SLNs: Soild lipid nanoparticles
HPOX: Hydroxybenzyl alcohol-incorporated polyoxalate
LPNs: Lipid–polymer hybrid nanoparticles
SiO_2_ NPs: Silica nanoparticles
MABAs: Muscarinic antagonist and β2 agonist properties
MMAD: Median mass aerodynamic diameter
SFD: Supercritical fluid
NCMPs: Nanocomposite microparticles
MF: Mometasone furoate
SX: Salmeterol xinafoate
SBS: Salbutamol sulphate
Nrf2: Dimethyl fumarate activator 2
BD: Budesonide
RES: Resveratrol
HA: Hyaluronic acid
HSPB5: Alpha B-crystallin
DEX: Dexamethasone
PVAX: Polyvinyl alcohol-containing copolyoxalate
HPH: High-pressure homogenization
SX: Salmeterol Xinafoate
FP: Fluticasone Propionate
TPGS: D-α-tocopherol polyethylene glycol 1000 succinate
BSA: Bovine serum albumin
FPF: Fine particle fraction
LPPs: Large porous microparticles
SLMs: Solid lipid microparticles
NCMPs: Nanocomposite microparticles
PRINT: Particle Replication In Non-wetting Templates
